# Voltage-Controlled Anodic Oxidation of Porous Fluorescent SiC for Effective Surface Passivation

**DOI:** 10.3390/nano10102075

**Published:** 2020-10-21

**Authors:** Kosuke Yanai, Weifang Lu, Yoma Yamane, Dong-Pyo Han, Haiyan Ou, Motoaki Iwaya, Tetsuya Takeuchi, Satoshi Kamiyama, Isamu Akasaki

**Affiliations:** 1 Department of Materials Science and Engineering, Meijo University, 1-501 Shiogamaguchi, Tenpaku-ku, Nagoya 468-8502, Japan; 203428035@ccmailg.meijo-u.ac.jp (K.Y.); 203428039@ccmailg.meijo-u.ac.jp (Y.Y.); han@meijo-u.ac.jp (D.-P.H.); iwaya@meijo-u.ac.jp (M.I.); take@meijo-u.ac.jp (T.T.); skami@meijo-u.ac.jp (S.K.); akasaki@meijo-u.ac.jp (I.A.); 2 Department of Photonics Engineering, Technical University of Denmark, DK-2800 Lyngby, Denmark; haou@fotonik.dtu.dk; 3 Akasaki Research Center, Nagoya University, Furo-cho, Chikusa-ku, Nagoya 464-8603, Japan

**Keywords:** fluorescent SiC, anodic oxidation, porous structures, photoluminescence, surface passivation

## Abstract

This study investigated the fabrication of porous fluorescent SiC using a constant voltage-controlled anodic oxidation process. The application of a high, constant voltage resulted in a spatial distinction between the porous structures formed inside the fluorescent SiC substrates, due to the different etching rates at the terrace and the large step bunches. Large, dendritic porous structures were formed as the etching process continued and the porous layer thickened. Under the conditions of low hydrofluoric acid (HF) concentration, the uniformity of the dendritic porous structures through the entire porous layer was considerably improved compared with the conditions of high HF concentration. The resulting large uniform structure offered a sizable surface area, and promoted the penetration of atomic layer-deposited (ALD) Al_2_O_3_ films (ALD–Al_2_O_3_). The emission intensity in the porous fluorescent SiC was confirmed via photoluminescence (PL) measurements to be significantly improved by a factor of 128 after ALD passivation. With surface passivation, there was a clear blueshift in the emission wavelength, owing to the effective suppression of the non-radiative recombination rate in the porous structures. Furthermore, the spatial uniformity of emitted light was examined via PL mapping using three different excitation lasers, which resulted in the observation of uniform and distinctive emissions in the fluorescent SiC bulk and porous areas.

## 1. Introduction

Generally, commercialized white light-emitting diodes (LEDs) comprise a blue LED and a yellowish phosphor layer, such as cerium-doped yttrium aluminum garnets [1,2,3,4,5,6]. High emission intensity can be achieved in these types of LED chips; however, the absence of a highly efficient red-light component means that the color-rendering index is relatively low. Therefore, other considerations have been proposed, e.g., white LEDs with a combination of red, green, and blue (RGB) LEDs [7,8], or the use of a near-ultraviolet (UV) LED to stimulate RGB phosphors [9]. However, the luminous efficiency of the green LEDs remains fairly poor, while the red phosphor can also absorb blue–green lights [2]; consequently, the popularization as a general lighting source is limited [8]. Fluorescent SiC is a promising phosphor for white LEDs due to its potential in achieving the requirements for low cost, a high color-rendering index, high emission intensity and efficiency, and high durability [5,10]. Despite SiC being an indirect bandgap semiconductor (3.0 eV), nitrogen (N) and boron (B) co-doped 6H–SiC can provide bright yellow–orange (around 578 nm) luminescence from donor–acceptor pair (DAP) emissions [5,10,11,12]. Moreover, porous structures fabricated in fluorescent SiC can emit simultaneous blue–green light, which is attributed to surface-defects-related emissions [12,13,14]. Passivation by atomic layer-deposited (ALD) dielectric films dramatically increased the emission intensity of the porous fluorescent SiC layer [15,16]. Therefore, it is expected that combining the yellow–orange emission from fluorescent SiC with the blue–green light in the porous structures will result in the emission of white light with a high color-rendering index [5,17].

In porous fabrication, anodic oxidation is a popular method for etching SiC, on account of its extreme chemical stability. Generally, the optical properties of porous SiC are largely determined by the morphological properties of pores [18,19], which are mainly affected by current density, compositions of both the etching solution, and the exposed surface (Si or C) during the anodic oxidation process [20,21]. A non-uniform distribution of porous structures was commonly observed as a consequence of porous depth when the anodic oxidation was conducted under a direct or pulsed constant-current mode [15,22]. Gautier et al. [23] found that the etching rate in 4H–SiC with a C-face is considerably higher than that of an Si-face, since an Si-terminated surface is more resistant than a C-face to electrolytic attack [24,25]. Furthermore, variations in pore size between 30 nm–50 nm have been reported according to different doping concentrations in SiC substrates [24]. However, the investigation of anodic oxidation in fluorescent SiC with high B and N dopants has rarely been reported. Presently, the current-controlled anodic oxidation method is mainly used to fabricate porous structures on common SiC substrates, despite the difficulty in realizing uniform porous structures throughout the entire layer. The homogenization of the porous structures is essential to obtaining both high emission intensity and sufficient surface passivation.

The present study conducted anodic etching on highly doped fluorescent 6H–SiC using a constant voltage-controlled method. The application of a high constant voltage can concentrate a large number of electrons/holes on the surface of fluorescent 6H–SiC substrates to provide stability for the anodic oxidation reaction process. This is expected to result in large and uniform porous structures for effective surface passivation using ALD–Al_2_O_3_ films. This study adjusted the hydrofluoric acid (HF) concentrations and anodic etching times to optimize the uniformity of the porous structures. The surface and cross-sectional morphologies were inspected using a scanning electron microscope (SEM). Homogenous structures were achieved throughout the whole porous layer, and the effect of step bunching on the surface morphologies was discussed. All samples were evaluated via both excitation-wavelength-dependent photoluminescence (PL) mappings and PL spectra characterization, conducted before and after ALD passivation. As a result, it was confirmed that the voltage-controlled anodic oxidation method is effective for improving the uniformity of porous structures in fluorescent SiC.

## 2. Materials and Methods

**Figure 1:**
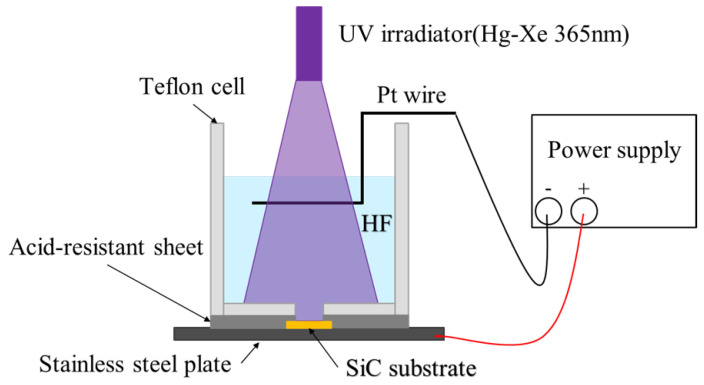
Schematic diagram of the experimental setup for the anodic oxidation process.

The fluorescent 6H–SiC substrates used in this experiment were grown via a closed sublimation growth process at 1700 °C, as previously reported by the authors’ research group [26]. The doping concentrations of N and B were confirmed by secondary ion mass spectroscopy to be 6.5 × 10^18^ cm^−3^ and 1.19 × 10^18^ cm^−3^, respectively. Nickel (Ni), titanium (Ti) and gold (Au) films were deposited on the backside (C-face) of the SiC samples to achieve ohmic contact. Firstly, an Ni film with a thickness of 100 nm was prepared using sputtering equipment (CFs-4EP-LL, Shibaura Mechatronics Co., Yokohama, Japan), before the annealing process, for 4 min at 900 °C. Then, Ti and Au films with thicknesses of 10 nm and 200 nm, respectively, were prepared using an electron beam evaporation system (EI-5, ULVAC Co., Chigasaki, Japan). Porous layers were formed on the SiC substrate via the anodic oxidation process using the apparatus detailed in Figure 1. The stainless-steel plate was used as the anode and the platinum wire was connected to the cathode. Conductive tape was used to attach the fluorescent samples to the stainless-steel plate. To prevent leakage of the HF acid, an acid-resistant sheet was inserted between the stainless-steel plate and the Teflon cell, and was screwed to the Teflon cell from the back of the stainless-steel plate. During the anodic oxidation, the samples were immersed in the HF solution and exposed under the UV irradiator (365 nm) using a mercury-xenon lamp. A constant voltage of 30 V was maintained using a DC voltage–current source/monitor (6241A, ADCMT Co., Saitama, Japan). By using the counter electrode and voltage source, the electric field in the space-charge layer of the fluorescent SiC substrate and hole concentration at the interface with HF solution could be controlled. Thereafter, the localized holes in the SiC surface could facilitate the rupture of bonds (oxidation step) and the dissolution of formed SiO_2_. The UV irradiation was applied to improve the distribution of hole concentration at the interface. UV irradiation not only provided energy to the HF solution, but also facilitated the anodic oxidation reaction process by exciting electrons from the valence band to the conduction band in the fluorescent SiC.

Porous fabrication was performed on three fluorescent SiC samples under identical growth conditions, as shown in Table 1. The porous samples were passivated by a 20 nm-thick Al_2_O_3_ film deposited by ALD (Model R200, Picosun, Espoo, Finland), followed by thermal annealing at 350 °C [15]. An atomic force microscope (AFM) (SPI3800N, Hitachi High-Tech Science Co., Tokyo, Japan) and an SEM (SU70, Hitachi Co., Tokyo, Japan) were used to characterize the surface morphologies of the epitaxial fluorescent SiC substrate and porous structures. To analyze the optical properties, PL measurements were performed with excitation provided by a 325 nm helium (He)-cadmium (Cd) laser (IK Series, KIMMON KOHA Co., Tokyo, Japan), while the emission signal was collected via a fiber spectrometer (USB4000, Ocean Optics Inc., Dunedin, USA). Finally, PL mapping (YWafer Mapper GS4, Ysystems Ltd., Tokushima, Japan) with excitation from three different lasers (325, 375, and 405 nm) confirmed the emission uniformity of the porous samples before and after ALD passivation.

## 3. Results and Discussion

**Figure 2:**
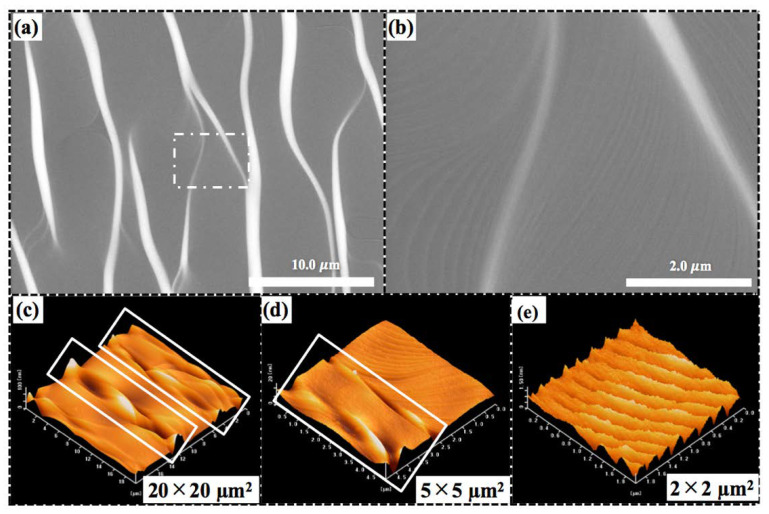
Planar scanning electron microscope (SEM) and atomic force microscope (AFM) images of the as-grown fluorescent substrate before anodic oxidation. Planar SEM images at (**a**) low-magnification and (**b**) high-magnification. (**c**–**e**) AFM images of the substrate at low, medium, and high magnification, respectively.

The planar SEM and AFM images of the as-grown fluorescent substrate used in this experiment were characterized before anodic oxidation. White curved belts and gray regions were observed in the SEM image, as presented in Figure 2a. The high-magnification SEM image within Figure 2b reveals that the step-bunching occurred in the gray region, which was comprised of various terrace widths. Typically, step-bunching might appear during the epitaxial growth on an Si-face substrate on account of the higher surface energy on Si faces compared with on C-faces, particularly at a high C/Si ratio [27]. As confirmed by the AFM measurements, the accumulation of multiple step bunches can yield curved hill-and-valley belts at some regions. AFM images at low and medium magnification are presented in Figure 2c,d, and the sections denoted by white rectangles correspond to the white areas in the SEM image [see Figure 2a]. Therefore, the terraces and large steps formed during epitaxial growth [28], and the terraces comprising uniform micro-step bunches are confirmed, as shown in Figure 2e. However, it should be noted that, the curved belts basically consist of both Si and C terminals [27], which might induce different anodic etching rates at the terrace and belt regions.

**Figure 3:**
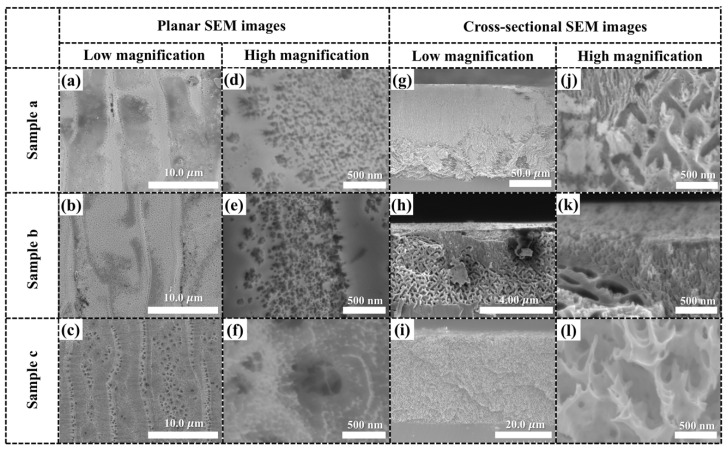
Planar and cross-sectional SEM images of the porous 6H–SiC samples after anodic oxidation process. Planar SEM images at (**a**–**c**) low-magnification and (**d**–**f**) high-magnification. Cross-sectional SEM images of samples a, b, and c, respectively, at (**g**–**i**) low-magnification and (**j**–**l**) high-magnification.

Both the planar and cross-sectional morphologies of the samples were inspected after anodic etching, as illustrated in Figure 3. It can be seen in Figure 3a–f that dense holes were particularly formed around the large, curved belts (large steps). Compared with the morphology of the terraces on the Si faces, the large, curved belts reacted more easily with the HF electrolyte than the terraces. This is due to the combination of Si and C terminals, whereas electrolytic etching is easier on C-terminated surfaces than Si-faces [24,25]. However, the terrace regions were also observed to contain multiple holes. The internal structures are visible in some regions, since those surface areas peeled off during etching. The sample c is seen to contain large pores, which were formed along the curved belts and terraces, as shown Figure 3c.

The thicknesses of the porous layer in samples a, b, and c are 110, 4.48, and 36.6 µm, respectively, as confirmed in Figure 3g–i. Thus, the average etching rates are 30.5, 213, and 10.2 nm/s for samples a, b, and c, respectively. Samples a and b were prepared by adjusting the etching duration, therefore it was predicted that a difference would be found in the thickness of the porous layer. However, the average etching rate of sample b was seven times higher that of sample a. It should be mentioned here that the HF concentration gradually decreased, and the UV irradiation effect also disappeared in the deep layer, resulting in a reduced etching rate under the same voltage supply. For sample c, the HF concentration decreased from 5% to 1%, which was much lower than that of the other samples. Therefore, the HF–SiC reaction rate decreased, as HF reduced the dissolution rate of the formed SiO_2_ during the oxidation steps. In addition, because of the higher resistance in 1% HF electrolyte, the injection current and vertical etching rate was reduced in sample c.

From the cross-sectional SEM images in Figure 3g–l, both large and small pores can be identified in every sample. It is considered that the small pores were initially formed and then expanded as the reaction proceeded. Sample b possesses a thin layer of porous structures with large pores evident under the terrace regions and small pores visible near the large, curved belts. As confirmed by the planar-view images, a large number of small pores were formed on the surface of the curved belts, allowing the HF electrolyte to penetrate easily into the deep layer of the fluorescent SiC. The large pores in the terrace region could expand quickly because HF was able to penetrate and concentrate the reaction in specific regions. As the etching progressed, the small holes in the lower part of the porous layer expanded obliquely, and the large dendritic porous structure became dominant. Larger porous structures were confirmed from the cross-sectional SEM image of sample a (dendritic structures) [see Figure 3g]. The porous structures in sample c were uniform throughout the whole porous layer from the top to the bottom, as shown in Figure 3i. This is attributable to the low anodic etching rate of fluorescent SiC in electrolyte with a low HF concentration, resulting in approximate rates in the horizontal and vertical direction. Overall, both a constant voltage supply and a low HF concentration were beneficial in achieving highly uniform porous structures in the fluorescent SiC substrate, which was associated with the uniformity of pores on the planar surface. This improvement can promote the ALD deposition for surface passivation.

**Figure 4:**
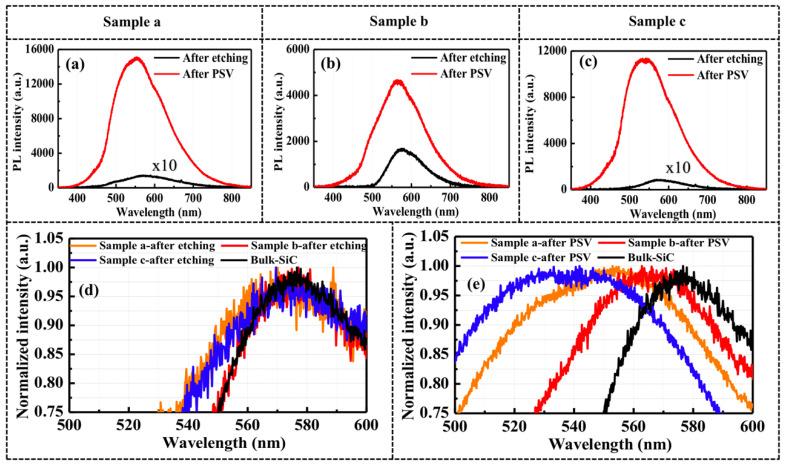
(**a**–**c**) Photoluminescence (PL) intensity of samples a, b, and c after anodic etching and after surface passivation (PSV); the intensity in samples a and c after etching is increased by a factor of 10. Normalized PL spectra in porous samples (**d**) before and (**e**) after surface passivation (PSV). The normalized PL spectrum of bulk fluorescent SiC is also plotted for comparison.

To verify the optical properties, PL measurements were carried out on the porous samples before and after surface passivation, as shown in Figure 4. The PL intensities of porous samples a and c are presented under 10 times magnification due to the weak emission after anodic oxidation process. A comparison of the PL intensities of samples a and c before and after anodic oxidation reveals a blueshift in the emission peaks, as shown in Figure 4a,c. It has been reported that emission in the shorter wavelength region is associated with oxygen vacancy defects, non-bridging oxygen hole centers, and C-related defects located near the interface of the porous SiC [12]. In this study, a large number of electrons/holes were injected into the fluorescent SiC substrate by the anodic oxidation under high voltage. As a result, this produced a considerable increase in the etching rate, the formation of a high-porosity layer, and a blueshift of the emission wavelength owing to the large porous surface area. Furthermore, the thicknesses of samples a and c are considerably larger than that of sample b, so the PL intensity was dominated by the emission from the porous layer. Nevertheless, degradation in the emission intensity of all three porous samples was observed due to the high density of non-radiative recombination centers on the porous surface. After surface passivation by ALD–Al_2_O_3_ films, samples a and c showed prominent PL enhancements of 104 and 128 times (average value of five points), respectively, which is significantly greater than the results previously reported elsewhere [12,15,22]. In sample b, the thin, porous layer caused the emission intensity to improve by a low factor of 2.7 following surface passivation, as shown in Figure 4b.

The normalized PL emission peaks in samples a, b, and c are plotted in comparison with the as-grown fluorescent SiC bulk, as shown in Figure 4d,e. The emission peaks of porous samples are dominated by the emission from the fluorescent SiC bulk due to the high non-radiative recombination rate in porous structures [15]. Nevertheless, it can be seen that the emission peaks in porous samples clearly blueshift after passivation, especially in sample c. This phenomenon is attributable to the sample’s uniformity and large, porous structure, which facilitates the effective coverage by ALD–Al_2_O_3_ films and the suppression of the non-radiative recombination rate. Basically, the pores formed in SiC substrate through anodic oxidation with a pulsed constant-current mode are around 30 nm–50 nm, and the size gradually decreases from the top, to the bottom areas [15]. If the porous structures are small through the whole layer, the ALD–Al_2_O_3_ can easily fill the pores in the top area, resulting in a limit of penetration depth to within 10 µm.

**Figure 5:**
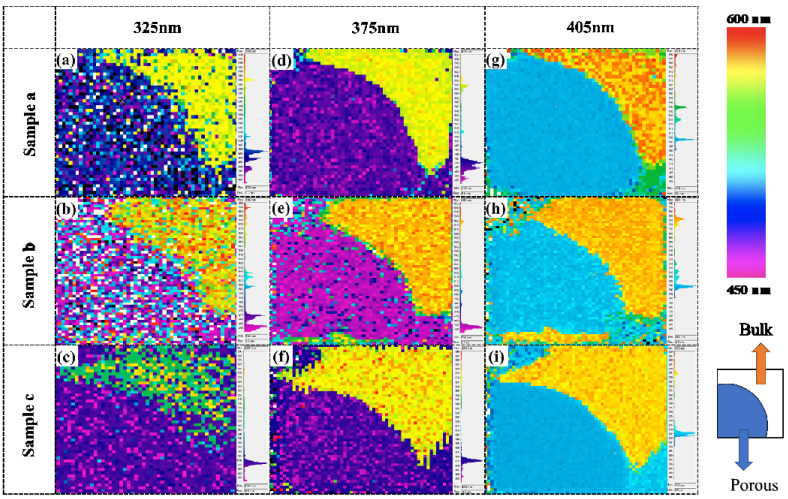
PL mapping of the porous fluorescent SiC samples a, b, and c, before passivation and under excitation, by (**a**–**c**) the 325 nm laser, (**d**–**f**) the 375 nm laser, and (**g**–**i**) the 405 nm laser, respectively. The color bar and configuration of the porous samples (5 × 5 mm^2^) are illustrated on the right.

PL mapping was conducted to inspect the uniformity and emission wavelength of each part of the porous samples before passivation, as depicted in Figure 5. Three different lasers with emission peaks at 405, 375, and 325 nm were used for excitation, respectively. In all samples, a clear separation of the peak wavelength between the fluorescent SiC and porous areas is evident. Firstly, the DAP emission peaks at around 578 nm in the fluorescent SiC bulk area are identified in all samples under three different excitations (>*E_g_*), as seen in the yellow–orange areas of the PL maps. Under the irradiation by the 325 nm and 375 nm lasers, the emissions from the porous area consisted of multiple peaks located at between 460 nm and 480 nm. The 405 nm laser penetrated deeper into SiC than the other two lasers; therefore, the observed emission wavelength in the PL maps contains the signal from the porous layer and from the underneath SiC bulk. Thus, as seen in Figure 5g–i, the emission peak in the porous area redshifts to ~500 nm. Moreover, the porous region of sample b (with its shallow porous depth) also indicates obvious emission, which was not identified in the PL spectra [see Figure 4d].

**Figure 6:**
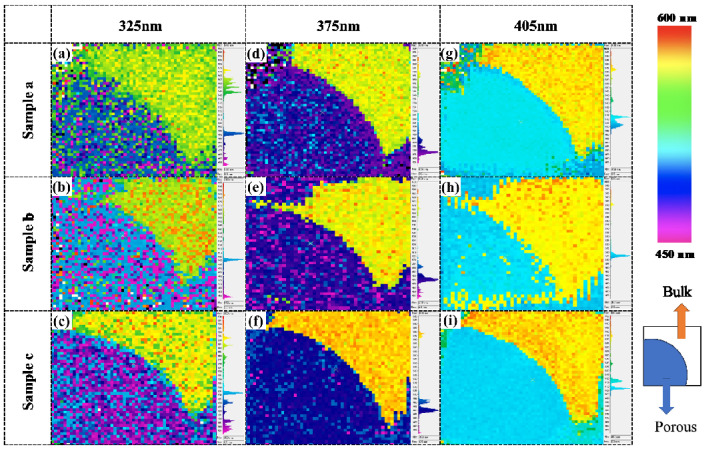
PL mapping of the porous fluorescent SiC samples a, b, and c, after passivation and under excitation, by (**a**–**c**) the 325 nm laser, (**d**–**f**) the 375 nm laser, and (**g**–**i**) the 405 nm laser, respectively. The color bar and configuration of the porous samples are illustrated on the right.

These results confirm that the porous layer can emit light in the short wavelength region, which is different from the yellow–orange emission in the bulk area. However, the low emission intensity in the porous layer before surface passivation resulted in the detection of a noise signal during measurement. After passivation with ALD–Al_2_O_3_, the porous samples were likewise characterized by PL mapping with excitation from three different lasers (405, 375, and 325 nm), as depicted in Figure 6. A fairly uniform emission distribution of all the porous samples is evident, with a clear boundary between the porous and fluorescent SiC bulk areas. This trend may be attributable to the enhanced emission intensity after passivation. These results verified that the constant-voltage-controlled anodic oxidation is effective in realizing uniform large, porous structures in fluorescent SiC, and improving the surface passivation by ALD–Al_2_O_3_ films.

## 4. Conclusions

In summary, we conducted a constant voltage-controlled anodic oxidation process to fabricate porous structures in fluorescent SiC substrates. Surface conditions including the terraces and large step bunches affected both the porous structures formed on the surface, and the internal porous structures formed in deep layers. Under low HF concentrations (1%), the porous structures developed into large dendritic structures, which promoted the surface passivation. After ALD surface passivation, the porous samples fabricated with 5% and 1% HF electrolyte were found to have a PL emission intensity enhanced by factors of 104 and 128, respectively. It is considered that the large surface area of the dendritic porous structures can facilitate the penetration of passivation into the deep layer. An analysis of the normalized PL spectra before and after passivation confirmed the emission in shorter wavelength range from the porous structures, owing to the effective suppression of non-radiative recombination rates in the porous structures. However, this phenomenon was not evident in the case of the thin, porous layer. To investigate the spatial uniformity of light emission, PL mapping was conducted using three lasers with different excitation wavelengths (i.e., different penetration depths). Consequently, uniform and discernable emissions were observed in both the fluorescent SiC bulk and porous areas, particularly following ALD passivation. These results confirm that the constant voltage-controlled anodic oxidation is effective for achieving uniform large dendritic pores throughout the entire porous layer. This finding has promise for achieving high passivation efficiency for fluorescent SiC-based LEDs.

## Figures and Tables

**Table 1:** Anodic oxidation conditions for porous samples a, b and c.

Sample Name	Thickness of Fluorescent SiC [µm]	Constant Voltage [V]	Hydrofluoric (HF) Concentration [%]	Anodic Etching Duration [s]	Thickness of Porous Layer [µm]	Etching Rate [nm/s]
a	200	30	5	3600	110	30.5
b	200	30	5	21	4.48	213
c	200	30	1	3600	36.6	10.2
